# Supplementary material for: Content-rich biological network constructed by mining PubMed abstracts
Source: BMC Bioinformatics. 2004 Oct 8;5:147. doi: 10.1186/1471-2105-5-147 (PMC528731; doi:10.1186/1471-2105-5-147)
Supplement: Additional File 2 — The original results of the above study (non-essential files are deleted to keep the file size under the limit set by BMC bioinformatics). [file 1471-2105-5-147-S2.bz2 › chilibotAdditionalFile2/dip50/468ID9660793E10524/html/CCL11_CXCR3.html]

 


 **CCL11** and **CXCR3** 
  
Found 87 abstracts in PubMed, retrieved 50.  
 

 What does Google say? 
 PDF only 
| .edu only 

---

**Interactive relationship** (e.g. stimulation, inhibition, etc)

**Neutral relationship**- Here, we report that although  **CCL11** , CCL24 and CCL26 are neither agonists nor antagonists of  **CXCR3** ,  **CCL11**  binds with high affinity to  **CXCR3** .  Ref: 12884299 Eur J Immunol, 2003
- N terminal domain of eotaxin  [ **CCL11** ]  3 is important for activation of CC chemokine receptor 3  [ **CXCR3** ] .  Ref: 12538912 Protein Eng, 2002
- This suggests that, in vivo,  **CXCR3**  may act as a decoy receptor, sequestering locally produced  **CCL11** .  Ref: 12884299 Eur J Immunol, 2003
- Eotaxin  [ **CCL11** ]  is a potent chemokine that acts via CC chemokine receptor 3  [ **CXCR3** ]  CCR3 to induce chemotaxis, mainly on eosinophils.  Ref: 12413953 Biochem Biophys Res Commun, 2002
- Eotaxin  [ **CCL11** ]  is a potent eosinophil chemoattractant and is a selective agonist for C C chemokine receptor 3  [ **CXCR3** ]  CCR3.  Ref: 11422151 Clin Exp Allergy, 2001
- Despite sharing considerable homology with the members of the monocyte chemoattractant protein MCP family, the CC chemokine eotaxin  [ **CCL11** ]   **CCL11**  has previously been reported to signal exclusively via the receptor CC chemokine receptor 3  [ **CXCR3** ]  CCR3.  Ref: 11559700 J Biol Chem, 2001
- Eotaxin  [ **CCL11** ] , eotaxin  [ **CCL11** ]  2 and eotaxin  [ **CCL11** ]  3 have been identified as ligands that only activate CC chemokine receptor 3  [ **CXCR3** ] .  Ref: 11286622 J Invest Dermatol, 2001
- Eotaxin  [ **CCL11** ]  is a potent inducer of eosinophil chemotaxis and was considered as a selective ligand of the CC chemokine receptor 3  [ **CXCR3** ]  CCR3, which is expressed on eosinophils, basophils, and Th2 lymphocytes.  Ref: 11264152 Blood, 2001
- Eotaxin  [ **CCL11** ] , an inducer of eosinophil migration and activation, exerts its activity by binding to CCR3, the C C chemokine receptor 3  [ **CXCR3** ] .  Ref: 12857386 J Biomol Screen, 2003
- Shikonin also did not inhibit monocyte chemoattractant protein 1 binding to CCR2 cells, eotaxin  [ **CCL11** ]  binding to CCR3 cells, interferon inducible T cell alpha chemoattractant binding to  **CXCR3**  cells and SDF 1alpha binding to CXCR4 cells.  Ref: 11360924 Int Immunopharmacol, 2001
- Furthermore, stimulation of cultured keratinocytes with eotaxin  [ **CCL11** ]  resulted in an increased 3H thymidine incorporation indicating a role of CC chemokine receptor 3  [ **CXCR3** ]  in epidermal proliferation and differentiation.  Ref: 11286622 J Invest Dermatol, 2001

**Non-interactive relationship** (e.g. studied together, co-existance, homology, etc.)

- In this study, we demonstrate that intranasal infection of BALB c mice with RSV A results in inducible expression of lung chemokines belonging to the CXC MIP 2 and IP 10  [ **CXCR3** ] , CC RANTES, eotaxin  [ **CCL11** ] , MIP 1beta, MIP 1alpha, MCP 1, TCA 3 and C lymphotactin families.  Ref: 11134301 J Virol, 2001
- Taking advantage of the humanized SCID mouse model grafted with autologous human skin, the ability of the chemokines IP 10  [ **CXCR3** ] , MDC, eotaxin  [ **CCL11** ] , and RANTES to stimulate cell recruitment was investigated.  Ref: 11238053 Am J Pathol, 2001
- These studies provide detailed information on the properties of MDC, eotaxin  [ **CCL11** ] , IP 10  [ **CXCR3** ] , and RANTES as chemotactic molecules in skin in vivo.  Ref: 11238053 Am J Pathol, 2001
- Distinct T cell subsets express different chemokine receptors, with type 2 cells mostly attracted by eotaxin  [ **CCL11** ] , MDC, TARC and I 309, and type 1 cells sensitive to IP 10  [ **CXCR3** ] , Mig, I TAC, RANTES and MIP 1beta.  Ref: 11876523 Arch Dermatol Res, 2002
- An eosinophil active chemokine, eotaxin  [ **CCL11** ] , and its receptor, C C chemokine receptor 3  [ **CXCR3** ] , are particularly attractive as novel targets of immunological intervention for the disease.  Ref: 12842759 Cytokine< MedlineTA>Cytokine, 2002
- In contrast, IP 10  [ **CXCR3** ]  is mostly expressed in T H 1 mediated diseases, and eotaxin  [ **CCL11** ]  expression seems to be specifically associated with lung diseases of a T H 2 cytokine profile.  Ref: 11295656 J Allergy Clin Immunol, 2001
- Xenograft rejection was associated with early intragraft transcript expression for monocyte chemotactic protein 1 MCP 1 3 to 5 days, IP 10  [ **CXCR3** ]  3 to 4 days and macrophage inflammatory protein 1alpha MIP 1alpha 3 to 5 days and subsequent expression of eotaxin  [ **CCL11** ]  days 4 to 10, MIP 1beta days 4 and 5 and regulated on activation, normal T cell expressed and secreted RANTES days 4 to 6 mRNA.  Ref: 12588649 Xenotransplantation, 2003
- In this study, we found that normal human keratinocytes activated with Th1 derived supernatant sup expressed early 6 12 h IP 10  [ **CXCR3** ]  CXCL10, MCP 1 CCL2, IL 8 CXCL8, and I 309 CCL1  [ **CCL11** ]  mRNAs and with slower kinetics 24 96 h, RANTES CCL5 and MDC CCL22 mRNAs.  Ref: 11590199 J Leukoc Biol, 2001
- Despite there being no difference in expression of CC chemokine receptor 3  [ **CXCR3** ] , the priming effect of RANTES and eotaxin  [ **CCL11** ]  was significantly enhanced in eosinophils from the patients.  Ref: 12797483 Eur Respir J, 2003
- Hence, eosinophil responses mediated by chemokines acting at CCR3 may be regulated by two distinct mechanisms the antagonistic effects of  **CXCR3**  ligands and the sequestration of  **CCL11**  by  **CXCR3**  expressing cells.  Ref: 12884299 Eur J Immunol, 2003
- The expression of IP 10  [ **CXCR3** ] , RANTES, and eotaxin  [ **CCL11** ]  were decreased in IL 12p40 mice and lung IL 6 expression was increased, all compared to controls.  Ref: 11784117 Exp Mol Pathol, 2002
- CKs fell into four categories a type 1 dominant gamma interferon inducible protein IP 10  [ **CXCR3** ] , monokine induced by INF gamma MIG, macrophage inflammatory protein 2 MIP 2, lipopolysaccharide induced chemokine LIX, rodent growth related oncogene homologue KP, macrophage inflammatory protein 1alpha MIP 1alpha and 1beta MIP 1beta, lymphotactin, b type 2 dominant eotaxin  [ **CCL11** ] , monocyte chemotactic protein 2 MCP 2 and 3 MCP 3, liver and activation regulated chemokine LARC, T cell activation protein 3 TCA 3, c type 1 and type 2 co dominant MCP 1, MCP 5, monocyte derived chemokine MDC, thymus and activation related chemokine TARC, C10, and d constitutive lungkine, secondary lymphoid tissue chemokine SLC, EBI1 ligand chemokine ELC, fractalkine, macrophage inflammatory protein 1gamma MIP1 gamma, and stromal cell derived factor 1alpha SDF1 alpha.  Ref: 11290568 Am J Pathol, 2001
- In particular, the eotaxin  [ **CCL11** ]  subfamily of chemokines and their receptor CC chemokine receptor 3  [ **CXCR3** ]  have emerged as central regulators of the asthmatic response.  Ref: 12589338 J Allergy Clin Immunol, 2003
- Type 1 chemokines CXC chemokine ligand CXCL 9 and CXCL10 monokine induced by IFN gamma and IFN gamma inducible protein of 10 kDa  **CXCR3**  ligands, and type 2 chemokines CC chemokine ligand CCL 11  [ **CCL11** ]  eotaxin  [ **CCL11** ]  CCR3 ligand, CCL17 thymus and activation regulated chemokine CCR4 ligand and CCL22 monocyte derived chemokine CCR4 ligand were readily detectable in the plasma of most participants.  Ref: 12407016 Int Immunol, 2002
- Consistent with these results, infected cells from WT mice preferentially migrated in response to IP 10  [ **CXCR3** ]   **CXCR3**  ligand, while those from GKO mice migrated in response to eotaxin  [ **CCL11** ]   **CCL11**  CCR3 ligand.  Ref: 12875978 Am J Pathol, 2003
- Biopsies were stained for CD68, CD3, elastase, eotaxin  [ **CCL11** ] , IP 10  [ **CXCR3** ] , MCP 1, MCP 3, and IL 8 and analyzed by NIH Image analyzer.  Ref: 12827410 Int J Colorectal Dis< MedlineTA>Int J Colorectal Dis, 2003
- This pattern was consistent with the early recruitment of macrophages MCP 1, MIP 1alpha, the influx of CD4 T cells MCP 1, MIP 1alpha, MIP 1beta, IP 10  [ **CXCR3** ]  and RANTES and the characteristic infiltrate of eosinophils eotaxin  [ **CCL11** ]  and RANTES associated with islet xenograft rejection.  Ref: 12588649 Xenotransplantation, 2003
- Recently, certain chemokines and chemokine receptors have been preferentially associated with the selective recruitment in vitro of type 1 T cells, such as IP 10  [ **CXCR3** ]  and its receptor  **CXCR3** , or type 2 T cells such as monocyte derived chemokine MDC and eotaxin  [ **CCL11** ]  and their receptors CCR4 and CCR3.  Ref: 11238053 Am J Pathol, 2001
- The chemokines eotaxin  [ **CCL11** ] , IP 10  [ **CXCR3** ] , lymphotactin, MCP 1, Mig, RANTES, and SDF 1 were examined in an additional 35 biopsies by RT PCR.  Ref: 11714650 Circulation, 2001
- Expression of RANTES, eotaxin  [ **CCL11** ] , MIP 1 alpha, MIP 1 beta, IP 10  [ **CXCR3** ] , and MCP 1 was upregulated by day 7 after infection.  Ref: 11853399 Virology, 2002
- Neutrophil recruitment was preceded by increased mRNA expression for tumor necrosis factor alpha, macrophage inflammatory protein 1alpha, macrophage inflammatory protein 2, macrophage chemotactic protein 1, and interleukin 6, but not interferon gamma, transforming growth factor ss, RANTES, eotaxin  [ **CCL11** ] , or IP 10  [ **CXCR3** ] .  Ref: 11141488 Am J Pathol, 2001
- To study mechanisms of progenitor cell mobilization in allergic reactions, we investigated fluctuations in the expression of the eotaxin  [ **CCL11** ]  receptor, CC chemokine receptor 3  [ **CXCR3** ]  CCR3, on CD34 cells from stable asthmatics following allergeni.e. antigen challenge.  Ref: 12871220 ImmunologyImmunology, 2001
- In parallel, we quantified plasma levels of IP 10  [ **CXCR3** ] , Mig, eotaxin  [ **CCL11** ]  and TARC.  Ref: 11423172 Hum Immunol, 2001
- The CXC chemokines such as IL 8, IP10  [ **CXCR3** ] , Mig, SDF 1 alpha or CC chemokines such as MCP 1, MIP 1 alpha, eotaxin  [ **CCL11** ] , RANTES have been frequently harvested from tumor tissues or the biological fluids of patients.  Ref: 12489485 Recenti Prog Med, 2002
